# Talar Allografts in Tibiotalocalcaneal Arthrodesis: A Salvage Approach for Complex Hindfoot Pathologies

**DOI:** 10.3390/jcm14082683

**Published:** 2025-04-14

**Authors:** Young Uk Park, Jae Ho Cho, Taehun Kim, Won-Tae Cho, Jinyoung Jun, Young Wook Seo

**Affiliations:** Department of Orthopedic Surgery, Ajou University Hospital, Ajou University School of Medicine, Suwon 16499, Gyeonggi-do, Republic of Korea; parkyounguk@gmail.com (Y.U.P.); cho@ajou.ac.kr (J.H.C.); realthkim@hanmail.net (T.K.); ccarius85@gmail.com (W.-T.C.); peapod0401@gmail.com (J.J.)

**Keywords:** hindfoot pathologies, talar allograft, tibiotalocalcaneal arthrodesis

## Abstract

**Background**: Tibiotalocalcaneal (TTC) arthrodesis using talar allografts has emerged as a viable surgical option for managing complex hindfoot pathologies, including post-traumatic avascular necrosis (AVN), infection-related complications, and failed total ankle replacement (TAR). These conditions present significant therapeutic challenges due to extensive bone loss and joint instability. Previous reports have focused on TTC arthrodesis using talar allografts, highlighting its potential to provide enhanced structural support. This study aims to further evaluate the efficacy and safety of this surgical approach by assessing union, clinical outcomes, and complications in a diverse patient population. **Methods**: This retrospective study reviewed 11 patients who underwent TTC arthrodesis with talar allograft between January 2020 and November 2022. The study cohort included patients with post-traumatic AVN, infection-related complications, and failed TAR. Preoperative and postoperative evaluations included X-rays, computed tomography scans, and functional outcome scores such as the Visual Analog Scale (VAS) and the Foot and Ankle Outcome Score (FAOS). **Results**: This study included 11 patients who underwent surgical treatment between January 2020 and November 2022, with a minimum follow-up of 24 months and a mean follow-up of 33.45 months (range, 24–50 months). Successful arthrodesis was observed in nine patients, yielding a success rate of 82%. Significant improvements in functional outcomes were noted, including marked reductions in pain and enhanced activity levels, as evaluated by VAS and FAOS scores. Two patients demonstrated radiographic nonunion (one tibiotalar, one subtalar), but both remained asymptomatic and did not require revision surgery. No other complications such as infection, wound issues, or thromboembolism were observed. Immediate postoperative radiographs confirmed appropriate allograft alignment and placement. **Conclusions**: TTC arthrodesis using structural talar allografts may be a viable and safe option for managing severe hindfoot pathology, potentially resulting in satisfactory fusion rates and clinical outcomes.

## 1. Introduction

Various conditions leading to talar collapse, including post-traumatic osteonecrosis, failed total ankle replacement (TAR), and severe infections such as osteomyelitis or septic arthritis, pose considerable challenges in orthopedic practice due to progressive joint destruction, intractable pain, and severely compromised mobility. The talus plays a pivotal role in ankle and hindfoot biomechanics, serving as the central load-transmitting structure between the leg and the foot.

Due to its unique anatomy—being largely covered by articular cartilage, lacking direct muscular attachments, and enclosed within a tight soft tissue envelope—the talus has a precarious vascular supply. This anatomical vulnerability renders it particularly susceptible to avascular necrosis (AVN), especially following traumatic events such as talar neck fractures or in the context of systemic conditions such as corticosteroid use, chronic alcohol intake, or autoimmune disease [1,2]. As AVN progresses, collapse of the talar dome can lead to joint incongruity, mechanical instability, and early-onset secondary osteoarthritis. As a result, managing talar collapse in advanced stages remains a significant clinical challenge due to the bone’s limited healing potential and the complex biomechanics of the hindfoot and ankle [3].

Several treatment modalities have been proposed to address talar pathology, including core decompression, vascularized bone grafts, and various arthrodesis techniques. However, these options often prove inadequate in cases of extensive bone loss or structural deformity [4]. Core decompression becomes ineffective once collapse occurs or subchondral integrity is compromised. Vascularized bone grafts, such as free fibular or medial femoral condyle grafts, offer osteogenic potential but require microsurgical expertise and may lack sufficient volume for reconstruction in cases of near-total talar loss [5,6,7,8]. Isolated ankle or subtalar arthrodesis may relieve pain but can result in malalignment, limb shortening, and adjacent joint degeneration when large segmental defects are present [9,10].

When conventional techniques fall short, more robust and anatomically congruent reconstructive options become necessary—particularly in cases involving large osseous defects, joint instability, and multiplanar deformity. Available alternatives include femoral head allografts, total talar prostheses, titanium cages, and antibiotic-loaded cement spacers. Among these, talar allografts and patient-specific implants have emerged as promising options due to their ability to restore structural and biomechanical integrity.

Talar allografts, in particular, offer distinct advantages. Their anatomical similarity to the native talus enables accurate restoration of hindfoot alignment and joint surface congruity. Unlike femoral head or tibial allografts, which often lack the complex curvature of the talar dome and subtalar articulation, talar allografts are shaped to replicate native morphology. This facilitates improved biomechanical load transfer and reduces the risk of graft malalignment or subsidence. Furthermore, unlike vascularized autografts, they avoid donor site morbidity and do not require microsurgical techniques. Although patient-specific 3D-printed implants offer tailored reconstruction, their high cost and limited accessibility may restrict their use in many clinical settings. Given this balance of anatomical fidelity, mechanical strength, and practicality, talar allografts represent a compelling option in the surgical management of severe talar deficiency.

Tibiotalocalcaneal (TTC) arthrodesis is widely accepted as a salvage procedure for end-stage hindfoot pathology. It is particularly indicated in cases of advanced talar bone loss due to trauma, AVN, or failed total ankle arthroplasty, where conventional fixation is insufficient. TTC fusion spans the ankle and subtalar joints, offering enhanced load sharing and biomechanical stability [11]. However, achieving successful arthrodesis and maintaining structural integrity in the setting of extensive bone deficits remains challenging.

In a systematic review and meta-analysis, Cifaldi et al. [10] reported that TTC arthrodesis with structural allografts achieved a 67.4% union rate and a 92.5% limb salvage rate in patients with large hindfoot bone defects, highlighting the clinical potential of this approach. Specifically, talar allografts have demonstrated favorable mechanical properties and anatomical compatibility in TTC arthrodesis [12].

Therefore, the purpose of this study was to evaluate the clinical and radiologic outcomes of TTC arthrodesis using talar allografts in patients with complex hindfoot pathologies. We hypothesized that this technique would achieve satisfactory fusion rates, functional improvement, and acceptable complication rates. By presenting a focused case series, this study aims to contribute to the evolving evidence base supporting the use of talar allografts in hindfoot reconstruction.

## 2. Materials and Methods

This study was approved by the Institutional Review Board.

### 2.1. Participants

This retrospective study included 11 patients with complex hindfoot pathologies, including talar osteonecrosis, failed TAR, and osteomyelitis, who underwent TTC arthrodesis using talar allografts between January 2020 and November 2022. The primary indications for surgery were post-traumatic osteoarthritis associated with talar avascular necrosis (AVN) in 7 cases, failed total ankle replacement (TAR) with concomitant AVN in 2 cases, chronic osteomyelitis involving the talus in 1 case, and infectious arthritis of the ankle joint in 1 case. Specifically, talar bone loss was considered unsuitable for traditional arthrodesis when preoperative imaging (CT or MRI) demonstrated ≥50% collapse or necrosis of the talar body, loss of structural continuity, or segmental bone defects that precluded stable fixation using conventional screw or plate constructs.

The cohort consisted of 6 males and 5 females, with a mean age of 48.2 years (range: 30 to 65 years). The mean duration of preoperative symptoms was 18.1 months (range: 6 to 42 months), highlighting the chronic nature and treatment-resistant profile of these conditions. The affected side was the right ankle in 7 cases and the left in 4 cases.

The Inclusion criteria were patients with significant talar bone loss that rendered traditional TTC arthrodesis techniques unsuitable. Patient demographics, including age, gender, duration of symptoms, and previous treatments, were recorded to ensure a comprehensive understanding of the study population (Table 1).

### 2.2. Surgical Technique

**Figure 1:**
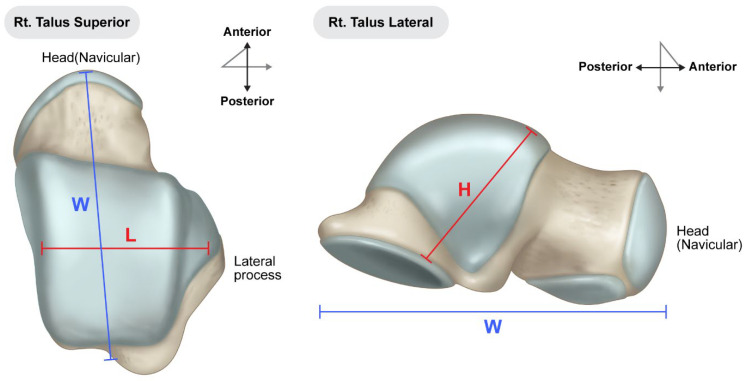
Preoperative measurements were performed using standard radiographs of the contralateral, unaffected talus to guide selection of an appropriately sized allograft. Key anatomical dimensions include: (L) mediolateral width measured across the talar body in the superior view; (W) anteroposterior length from the talar head to the posterior margin in the superior view; (H) maximum vertical height from the apex of the talar dome to the posterior facet in the lateral view. These measurements were used to select the most anatomically compatible allograft from the national tissue bank for each patient.

Preoperative surgical planning was initiated through detailed CT and plain radiographic evaluation of the contralateral, unaffected talus to determine the most appropriate size and shape of the allograft. This templating process included measurement of the maximum vertical height from the apex of the talar dome to the posterior facet (on the lateral view), the anteroposterior length of the talar head (on the superior view), and the maximal mediolateral width (Figure 1). These dimensions were used to estimate the optimal talar geometry. Additionally, patient-specific factors such as body height, body mass, and limb laterality were considered to enhance size matching accuracy. These preoperative measurements were then compared against a catalog of available cadaveric talus allografts from the national tissue bank to identify a suitable donor match. Once selected, the appropriate-sized fresh–frozen talus allograft was reserved and procured for intraoperative use. As of 2023, the cost of an allogeneic talus through the Korea Public Tissue Bank is approximately EUR 400, whereas, a femoral head allograft suitable for talar reconstruction costs approximately EUR 480.

**Figure 2:**
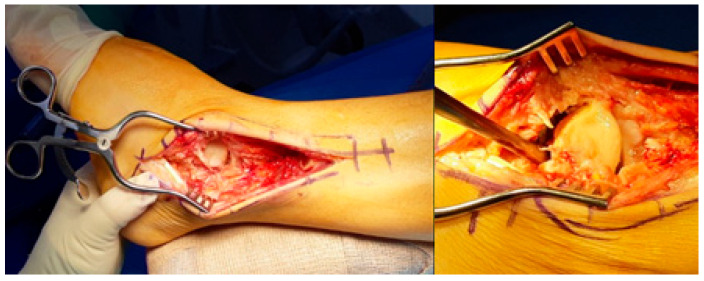
Employing a traditional lateral transfibular approach, necrotic and unstable talus was removed.

Surgical intervention was performed via a traditional lateral transfibular approach, which offers excellent exposure of both the ankle and subtalar joints (Figure 2). After a careful skin incision and subperiosteal dissection, the distal fibula was osteotomized and removed to create an unobstructed surgical field. Preoperative MRI and CT imaging were reviewed to delineate the extent of avascular necrosis and guide the resection boundaries. Intraoperatively, the extent of necrotic bone was assessed visually and mechanically, based on discoloration, fragmentation, and the absence of punctate bleeding—commonly referred to as the “paprika sign”—upon burring. Nonviable portions of the talus were excised using osteotomes and rongeurs, while care was taken to preserve any residual viable structures and soft tissue attachments, particularly around the sinus tarsi and deltoid ligament, to maintain local vascular supply.

**Figure 3:**
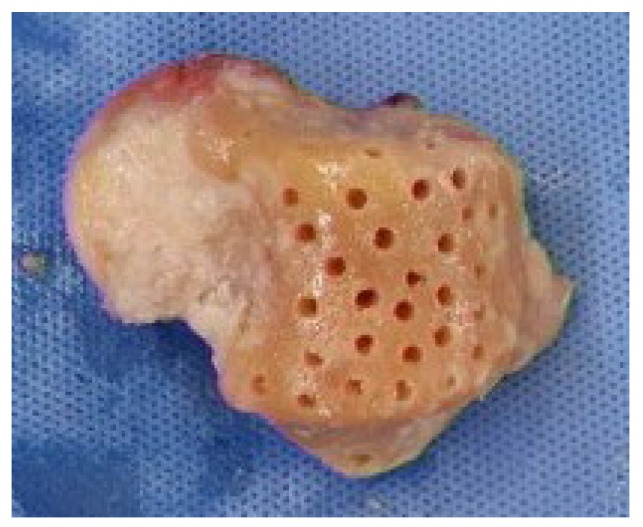
Meticulously sized fresh–frozen talus allograft, prepared to match the contralateral talus, was employed. The allograft was carefully tailored, and all cartilage was excised to ensure a precise fit. Drilling was conducted using a K-wire into the subchondral bone of the talus body to promote graft incorporation.

Following debridement, a fresh–frozen cadaveric talus allograft was thawed and carefully shaped to replicate the anatomy of the resected native talus. All articular cartilage was removed to create a raw cancellous surface, facilitating graft incorporation. K-wire drilling into the recipient talar body was performed to promote revascularization and osteointegration Bone marrow aspiration was routinely performed in all patients to enhance osteogenic potential and support graft incorporation (Figure 3). Approximately 20 mL of bone marrow aspirate was harvested from the anterior iliac crest and applied to the graft-host interface. The aspirate was concentrated and introduced at the graft-host interface to enhance osteogenic potential.

**Figure 4:**
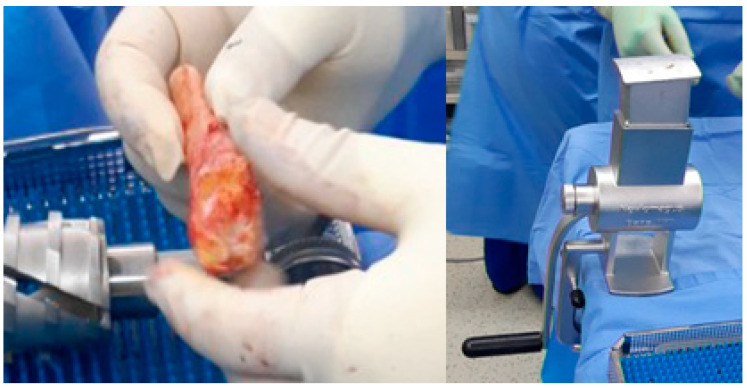
The remaining talus head and fibular bone were prepared using a bone mill and utilized as an additional bone graft to augment structural integrity.

**Figure 5:**
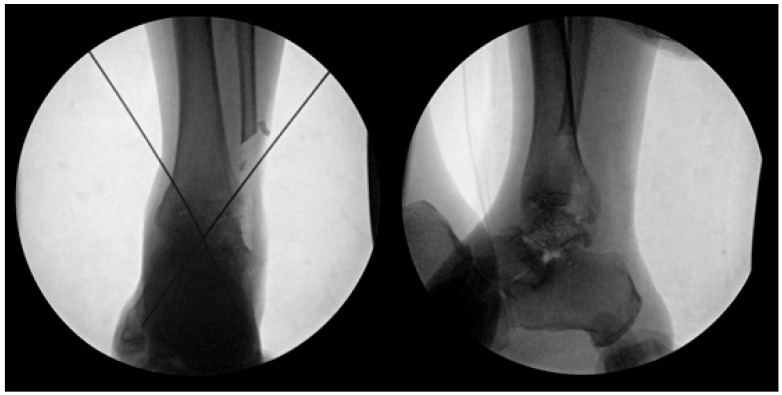
The prepared talus allograft was then inserted into the defect and initially stabilized using K-wires.

**Figure 6:**
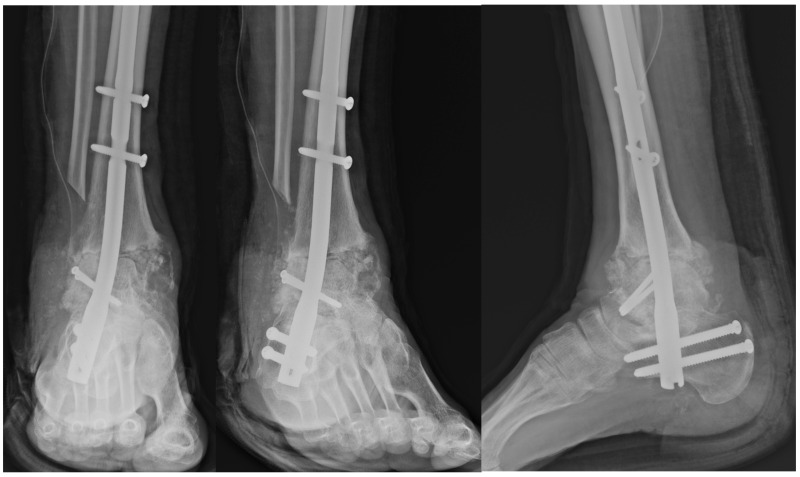
Postoperative radiograph demonstrating fixation of the talar allograft to the residual talar head using two 3.5 mm cannulated screws for structural stability.

Autologous bone grafts were also utilized intraoperatively. The excised fibular shaft and resected talar fragments were morselized using a bone mill and used as local cancellous graft material to fill any residual defects and augment the fusion site (Figure 4). The shaped allograft was inserted into the prepared talar void and temporarily stabilized with multiple Kirschner wires (Figure 5). Final fixation was achieved with a retrograde intramedullary (IM) nail system (Stryker^®^ T2 Ankle Arthrodesis Nail), inserted from the plantar surface of the calcaneus through the talus into the tibia. Additional fixation with two 3.5 mm cannulated screws targeting the residual talar head or anterior portion of the graft was applied in cases where enhanced rotational or axial stability was deemed necessary (Figure 6).

In cases involving active or prior infection—such as chronic osteomyelitis or septic arthritis—a two-stage surgical protocol was followed. The initial procedure consisted of thorough surgical debridement of infected and necrotic tissues, followed by implantation of an antibiotic-loaded talus-shaped cement spacer. The spacer was customized to approximate the native talar geometry and maintain joint space. Infection control was confirmed through clinical resolution of signs and symptoms, normalization of inflammatory markers, and intraoperative frozen section analysis demonstrating ≤5 white blood cells per high-power field. Only after clear evidence of infection resolution was the definitive TTC arthrodesis with talar allograft performed using the same surgical technique as described above.

### 2.3. Postoperative Care

**Figure 7:**
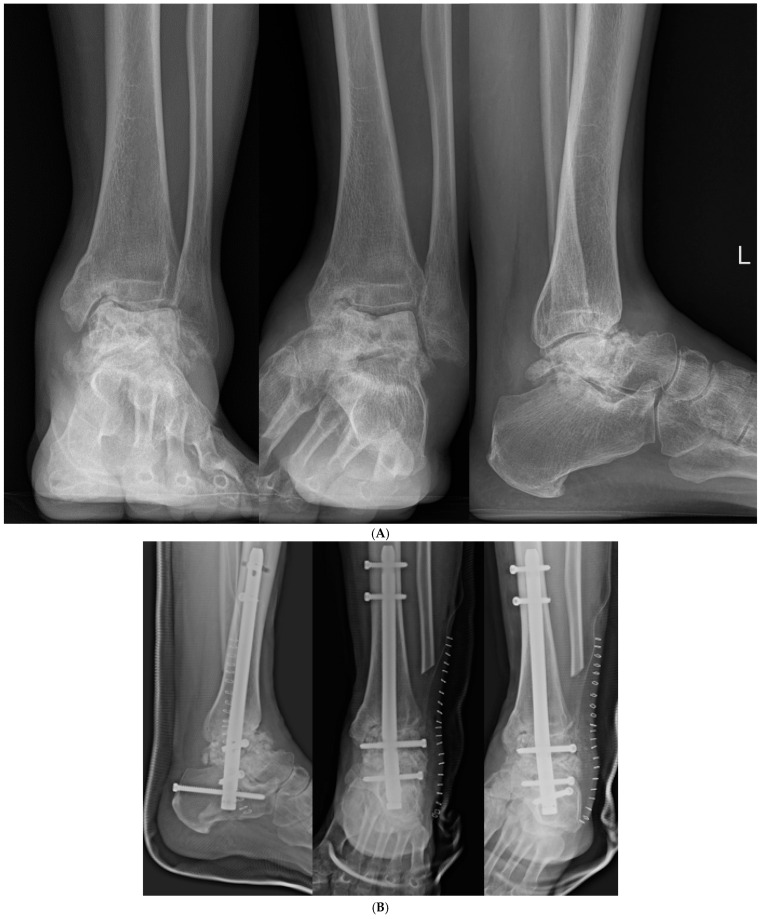
(**A**) Preoperative and (**B**) postoperative X-ray Findings.

**Figure 8:**
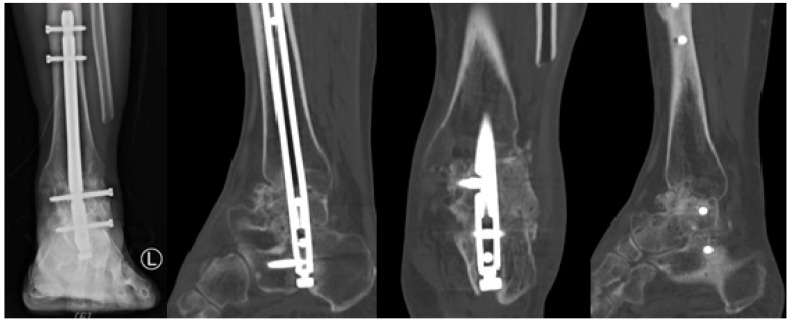
Two-Years Postoperative X-Ray and Computed Tomography Findings.

Postoperatively, patients were immobilized in a non-weight-bearing cast for 4 weeks to ensure initial stabilization and healing. Following this initial immobilization period, patients were transitioned to wearing controlled ankle motion boots for an additional 4 weeks to maintain non-weight-bearing status. After the total 8-week period of non-weight-bearing, patients began progressive, tolerable weight-bearing activities. Full weight-bearing was permitted at 12–16 weeks post-surgery, depending on individual patient progress and radiographic evidence of graft incorporation and arthrodesis (Figure 7 and Figure 8).

### 2.4. Outcome Measures

Follow-up assessments were scheduled at 2, 4, 8 weeks, as well as at 3, 4, 6 and 12 months post-surgery. After the first year, annual follow-ups were conducted to monitor long-term outcomes. These assessments included clinical evaluations and imaging studies using X-rays and computed tomography (CT) scans to monitor graft incorporation, arthrodesis status, and overall clinical outcome. Graft incorporation and fusion were evaluated using clearly defined radiographic criteria, including: (1) the presence of continuous trabecular bone bridging across the graft–host interface, (2) the absence of radiolucent lines, and (3) signs of progressive bone remodeling and graft integration over time on serial X-ray and CT imaging. Clinical signs such as the absence of pain and physical stability at the arthrodesis site were also considered. In cases of unexplained pain or equivocal imaging findings, further evaluation was performed using laboratory tests—including ESR, CRP, and WBC counts—to screen for infection. Additional imaging such as MRI was selectively used to assess potential graft necrosis or deep-seated infection. However, no cases in this study exhibited clinical or radiologic evidence of subclinical infection or sterile graft failure. Functional outcomes were assessed using the Visual Analog Scale (VAS) and the Foot and Ankle Outcome Score (FAOS).

### 2.5. Statistics

Statistical analyses were conducted using the Wilcoxon signed-rank test to compare preoperative and postoperative functional scores. A *p*-value of < 0.05 was considered statistically significant. Data were analyzed using SPSS software (version 26.0; IBM Corp., Armonk, NY, USA), and all statistical analyses were performed by the authors. No independent statistician was involved.

## 3. Results

### 3.1. Demographics

This study included a total of 11 patients who underwent tibiotalocalcaneal (TTC) arthrodesis with a femoral head-derived bulk talar allograft between January 2020 and November 2022. All patients were followed for a minimum duration of 24 months postoperatively, with a mean follow-up period of 33.5 ± 7.2 months (range, 24 to 50 months), allowing for adequate evaluation of clinical and radiologic outcomes.

The mean age at the time of surgery was 46.3 ± 10.1 years, ranging from 25 to 63 years. The cohort consisted of seven male and four female patients. In terms of laterality, 6 cases involved the right ankle and five cases involved the left. Diagnoses leading to surgical intervention included post-traumatic osteoarthritis (OA) associated with talar avascular necrosis (AVN) in seven patients (63.6%), failed total ankle arthroplasty (TAR) with concomitant AVN in 2 patients (18.2%), chronic osteomyelitis of the talus in 1 patient (9.1%), and infectious arthritis involving the tibiotalar and subtalar joints in 1 patient (9.1%).

All procedures were performed using the transfibular approach, which provides broad exposure of the ankle and subtalar joints while allowing for resection of the distal fibula to facilitate graft placement and nail insertion. Fixation was uniformly achieved using a retrograde intramedullary (IM) nail across the ankle and subtalar joints, ensuring axial alignment and compression at the fusion interfaces. In most cases, additional bone graft material was packed around the allograft interface to enhance union, although no structural autograft was used.

All patients underwent successful reconstruction without intraoperative complications. Table 1 summarizes the demographic and clinical details of each patient, including age, sex, diagnosis, surgical method, arthrodesis type (primary or secondary), follow-up duration, union status, smoking history, and complications (Table 1).

### 3.2. Union

Successful bony union was achieved in 9 out of 11 patients (81.8%), as confirmed through serial radiographs and computed tomography (CT) scans obtained between 3 and 12 months postoperatively. Union was defined radiographically by the presence of continuous trabecular bridging across the fusion site and the absence of implant loosening or hardware failure. On CT imaging, union was defined as complete bony continuity without any radiolucent gap at the host–graft interface. Among the two patients who did not achieve complete union, one exhibited tibiotalar nonunion, and the other demonstrated subtalar nonunion.

A detailed case analysis of the two patients with nonunion revealed several patient-related and procedural factors that may have contributed to the outcome. The patient with tibiotalar nonunion was a 62-year-old male who underwent TTC arthrodesis as a revision procedure following failed total ankle arthroplasty (TAR). Preoperatively, he presented with implant loosening and advanced post-TAR arthritis of the right ankle. His medical history was notable for multiple risk factors known to impair bone healing, including long-standing diabetes mellitus (>10 years), alcoholic liver cirrhosis for 12 years, and a 20 pack-year smoking history. Additionally, the revision nature of the surgery and compromised bone quality at the fusion site may have further increased the risk for nonunion.

The second case involved a 35-year-old male with subtalar nonunion. He had a history of avascular necrosis of the talus, a previous talar neck fracture, and a pre-existing nonunion at the same location. The surgery was performed on the left ankle. Despite radiographic evidence of incomplete osseous bridging at the subtalar joint on follow-up CT imaging, the patient remained entirely asymptomatic and reported no pain or functional limitations at the two-year follow-up. A long-standing smoking history (0.4 packs per day for 40 years) was considered a potential contributing factor affecting graft incorporation.

Notably, neither patient with radiographic nonunion reported any postoperative symptoms or discomfort. Both experienced substantial reductions in pain and improvements in functional status compared to their preoperative baseline. Their Foot and Ankle Outcome Scores (FAOS) and Visual Analog Scale (VAS) scores showed marked improvement. In light of the absence of clinical symptoms, maintained limb alignment, and stable implant positioning, additional procedures such as revision arthrodesis or bone grafting were not required during the follow-up period.

These findings underscore the importance of individualized risk assessment in complex TTC arthrodesis using structural allografts. While radiographic union remains a critical marker of surgical success, clinical outcomes and patient-reported measures should be equally considered when evaluating the need for further intervention.

### 3.3. Functional Outcomes

In all patients, a statistically significant reduction in pain levels was observed following surgery when compared to preoperative assessments. The mean Visual Analog Scale (VAS) score decreased markedly from 5.4 ± 1.2 before surgery to 1.3 ± 0.6 at the final two-year follow-up (*p* < 0.02), indicating a substantial improvement in subjective pain perception. This reduction was consistent across the cohort, regardless of the underlying pathology or comorbidities, suggesting that TTC arthrodesis using talar allografts effectively alleviates chronic pain in cases of severe talar bone loss.

In addition to pain relief, patients demonstrated notable functional recovery as reflected in all subscales of the Foot and Ankle Outcome Score (FAOS). The FAOS pain subscale improved from a mean of 45.2 ± 9.1 preoperatively to 72.3 ± 10.8 at two years postoperatively. Similarly, the symptoms subscale increased from 48.6 ± 8.7 to 79.4 ± 9.5, and the activities of daily living (ADL) score improved from 50.1 ± 10.2 to 75.7 ± 12.4. The sports and recreation function subscale, which is often more difficult to restore in complex foot and ankle conditions, improved from 30.4 ± 11.3 to 60.8 ± 13.7. Lastly, the quality of life (QOL) subscale rose from 34.9 ± 9.8 to 74.5 ± 12.1. All changes across FAOS domains were statistically significant (all *p* < 0.04), underscoring the comprehensive benefit of the procedure on both functional performance and overall well-being (Table 2).

Regarding postoperative safety, no short-term perioperative complications were observed during hospitalization or the early recovery period. Specifically, there were no cases of superficial or deep infection, soft tissue necrosis, wound dehiscence, or deep vein thrombosis (DVT). Although two cases of radiographic nonunion were noted during follow-up, neither patient experienced clinical symptoms nor required secondary surgical intervention. The absence of additional systemic or local complications further supports the viability and safety of using bulk talar allografts in TTC arthrodesis for this challenging patient population.

## 4. Discussion

The most important finding of this study is that TTC arthrodesis using talar allografts achieved a high union rate of 81.8% in patients with complex ankle pathologies, demonstrating its efficacy in providing robust structural support and promoting successful arthrodesis despite significant risk factors. Furthermore, the patients who encountered nonunion did not experience notable discomfort in daily activities and did not require additional surgical intervention.

TTC arthrodesis is a representative method for addressing severe hindfoot pathologies. It is widely used to address various ankle and subtalar joint conditions, including OA, Charcot arthropathy, AVN, failed ankle arthroplasty, and severe deformities. Additionally, TTC arthrodesis has proven effective as salvage therapy in patients with severe hindfoot trauma [13,14]. It serves as an excellent option in patients with poor bone stock, severe deformity, or when accompanied by subtalar joint arthritis. The structural support provided by TTC arthrodesis can effectively address the complexities associated with these challenging conditions, restoring stability and function to the affected limb [15]. Recent studies have also reported favorable outcomes with minimally invasive TTC arthrodesis using retrograde intramedullary nails. Biz et al. demonstrated 100% fusion and improved functional scores using a percutaneous approach, with reduced soft tissue morbidity and early recovery. However, this technique may be limited in cases with severe bone loss or deformity, where structural allografts remain essential [16].

Various fixation methods have been utilized in tibiotalocalcaneal (TTC) arthrodesis, including crossed screws, plate constructs, and retrograde intramedullary (IM) nails [14,15,17]. Each method presents distinct biomechanical characteristics and technical considerations. Screw fixation, while relatively simple and cost-effective, may not provide sufficient axial and rotational stability in cases involving poor bone stock or extensive talar bone loss. Plate fixation offers enhanced rigidity and compression but often requires extensive soft tissue dissection, increasing the risk of wound complications and hardware prominence. Retrograde IM nailing has emerged as a preferred technique for TTC arthrodesis in patients with complex hindfoot pathology, particularly in cases requiring salvage reconstruction. Budnar et al. [18] reported that TTC arthrodesis using retrograde IM nails offers several advantages. These include a minimally invasive surgical approach with reduced soft tissue disruption, superior axial and torsional stability, and the potential to promote earlier postoperative weight-bearing due to load-sharing properties of the nail construct. Additionally, the intramedullary position of the nail helps maintain alignment through the mechanical axis of the limb, contributing to more favorable biomechanical loading conditions across the fusion site. From a clinical perspective, these biomechanical and technical benefits translate into improved union rates, shorter immobilization periods, and reduced complication risks in selected patient populations. In particular, in cases involving allograft reconstruction, where achieving stable fixation is critical to graft incorporation, the use of a retrograde IM nail offers a reliable and structurally sound option. As such, it plays a pivotal role in optimizing outcomes following TTC arthrodesis in the setting of large segmental talar defects.

TTC arthrodesis and the use of structural allografts as salvage therapy involving the lower extremities have been described [19,20,21,22]. In patients with large bone defects undergoing TTC arthrodesis, a meta-analysis that included 175 patients reported that the femoral head was used as an allograft in 90% of the patients, while the distal tibia was used in 9% [10]. The overall union rate in this study was 67.4% [10]. The femoral head is a commonly used allograft in orthopedic surgery, with reported arthrodesis rates ranging from 50% to 93%, demonstrating reasonable results [21]. However, a significant drawback is the potential for collapse through the graft itself [23,24]. This highlights the need for careful allograft material selection for TTC arthrodesis, balancing the benefits of structural support with the risk of graft failure [10]. In contrast, DeFontes et al. [12] reported the first study describing the use of a whole talar body allograft for TTC arthrodesis, with six procedures performed and initial success observed. Preliminary results indicated a high arthrodesis rate with minimal complications, and thus far, no patients experienced nonunion, deep infection, or required amputation. This technique offers potential benefits compared to bulk femoral head allografts, such as providing a better anatomical fit and a more rigid structural support [24]. In the present study, a union rate of 82% was achieved, with 9 of the 11 patients experiencing successful fusion. Tibio-talar and subtalar nonunions were noted, but no further surgical intervention or complications were reported. Recent advances such as 3D printing have enabled the development of patient-specific implants for complex bone defects. Brachet et al. highlighted its potential in orthopedic reconstruction. While still emerging, these technologies may complement or offer future alternatives to structural allografts [25]. In comparison to other graft types such as femoral head or autografts, talar allografts offer structural congruence with the native talus and improved load distribution in complex hindfoot reconstruction. While bone marrow aspirate and autologous morselized grafts were used adjunctively in this study to support osteogenesis, they were not utilized as primary grafting materials. Thus, the fusion outcomes are primarily attributable to the structural role of the talar allograft, although the exact contribution of adjuncts cannot be fully separated. Nevertheless, the absence of a control group using alternative graft types (e.g., femoral head or autograft) limits direct comparative evaluation. Future studies incorporating control cohorts or randomized comparative designs will be essential to delineate the independent effectiveness of talar allografts in TTC arthrodesis.

Nonunion remains a significant concern in TTC arthrodesis, particularly in patients with comorbid conditions [26,27]. Risk factors such as diabetes mellitus, smoking, and poor bone quality substantially increase the likelihood of nonunion [26,28,29]. Pitts et al. [29] reported a nonunion rate of 28.7%, with increased rates observed in patients with Charcot arthropathy, non-traumatic OA, diabetes, and chronic kidney disease. Additionally, patients aged >60 years demonstrated a significantly higher risk of postoperative infection. Previous studies have indicated that patients with diabetes experience impaired bone healing due to neurological and vascular impairment, resulting in lower arthrodesis rates and higher complication rates [30]. Diabetes, frequently accompanied by other systemic illnesses, significantly exacerbates the risk of suboptimal surgical outcomes [31]. Hyperglycemia and insulin deficiency in patients with diabetes detrimentally affect bone integrity by hindering osteoblast activity and collagen synthesis. These metabolic issues, frequently associated with Charcot neuroarthropathy, contribute to poor arthrodesis rates in hindfoot and midfoot procedures [32,33]. In the present study, despite achieving a high overall union rate, two of the eleven patients who underwent TTC arthrodesis with talus allografts experienced nonunion. These nonunions might have been associated with comorbidities such as diabetes, liver cirrhosis, smoking, and failed TAR. Addressing these risk factors preoperatively through rigorous management of diabetes and smoking cessation programs may enhance arthrodesis rates and overall outcomes.

Most studies reporting TTC arthrodesis outcomes have revealed improvements in FAOS and pain scores [34]. However, reports regarding other outcomes and complications vary widely, with nonunion and complication rates ranging from 3.4% to 48% [34,35]. In this study, no major complications or infections were observed, and immediate postoperative radiographs confirmed proper alignment and placement of the allograft, with the final follow-up demonstrating stable union and satisfactory functional recovery.

The present study had several limitations. First, the sample size was small, which significantly limits the statistical power and generalizability of the findings. While the outcomes are encouraging, they must be interpreted with caution given the heterogeneity and complexity of the patient population. Accordingly, this study should be regarded as an exploratory case series that provides preliminary insight into the feasibility and potential utility of talar allografts, rather than definitive clinical evidence. Second, the retrospective design may introduce selection bias and limits the ability to control for confounding variables. Third, the study population included a small subset of patients with a history of infection (osteomyelitis or infectious arthritis), who underwent a staged surgical protocol involving initial debridement and placement of an antibiotic spacer. These cases differ significantly from the non-infectious cohort in terms of biological environment and surgical complexity, potentially limiting the comparability of outcomes across the entire sample. In addition, the limited number of patients precluded the use of multivariate statistical analyses, such as logistic regression, to identify independent predictors of nonunion or other complications. Furthermore, graft revascularization—an important determinant of long-term viability—was not directly evaluated, as advanced perfusion imaging (e.g., SPECT/CT or contrast-enhanced MRI) was not routinely performed. Future prospective studies with larger cohorts, standardized surgical protocols, and advanced imaging modalities are needed to validate these findings and better characterize the clinical performance of talar allografts in complex hindfoot reconstructions.

## 5. Conclusions

The combination of an intramedullary (IM) nail and talar allograft in tibiotalocalcaneal (TTC) arthrodesis may represent an effective salvage strategy, particularly for patients with extensive bone defects. The use of a talar allograft in these cases may contribute to improved union rates and reduced complications, including graft collapse. This technique may serve as a promising therapeutic option for managing severe bone loss associated with talar collapse in appropriately selected patients undergoing TTC arthrodesis. However, further studies with larger cohorts are needed to confirm these preliminary findings.

## Figures and Tables

**Table 1:** Demographics and clinical details of patients undergoing TTC arthrodesis with talar allografts.

Patient	Age/Sex	Cause of Talar Collapse	Arthrodesis (Primary or Secondary)	Follow-Up Period (Months)	Union Status	Smoking Hx.	Complications
1	52/M	Post-traumatic OA (AVN talus)	Primary	50	Union	None	None
2	35/M	Post-traumatic OA (AVN talus)	Primary	48	Nonunion(subtalar)	0.4 pack × 15 Y	None
3	50/M	Post-traumatic OA (AVN talus)	Primary	40	Union	0.5 pack × 30 Y	None
4	25/M	Post-traumatic OA (AVN talus)	Primary	36	Union	None	None
5	62/M	Failed TAR and AVN talus	Secondary	36	Nonunion(tibiotalar)	0.5 pack × 35 Y	None
6	27/M	Osteomyelitis	Secondary	30	Union	None	None
7	59/F	Failed TAR and AVN talus	Secondary	30	Union	None	None
8	67/F	Infectious arthritis	Secondary	24	Union	None	None
9	56/M	Post-traumatic OA (AVN talus)	Primary	26	Union	1.0 pack × 30 Y	None
10	64/M	Post-traumatic OA (AVN talus)	Primary	24	Union	1.0 pack × 45 Y	None
11	74/M	Post-traumatic OA (AVN talus)	Primary	24	Union	None	None

**Table 2:** Preoperative and final follow-up functional outcome scores (VAS and FAOS Subscales).

Score Type	Preoperative (Mean ± SD)	Final Follow-Up (Mean ± SD)	*p*-Value
VAS	5.4 ± 1.2	1.3 ± 0.6	<0.02
FAOS–Pain	45.2 ± 9.1	72.3 ± 10.8	<0.04
FAOS–Symptoms	48.6 ± 8.7	79.4 ± 9.5	<0.04
FAOS–ADL	50.1 ± 10.2	75.7 ± 12.4	<0.04
FAOS–Sports	30.4 ± 11.3	60.8 ± 13.7	<0.04
FAOS–QOL	34.9 ± 9.8	74.5 ± 12.1	<0.04

Abbreviations: VAS, visual analog scale; FAOS, foot and ankle outcome score; ADL, activities of daily living; QOL, quality of life.

## Data Availability

The original contributions presented in this study are included in the article. Further inquiries can be directed to the corresponding author(s).

## References

[1] Parekh S.G., Kadakia R.J. (2021). Avascular necrosis of the talus. JAAOS-J. Am. Acad. Orthop. Surg..

[2] Adelaar R.S., Madrian J.R. (2004). Avascular necrosis of the talus. Orthop. Clin..

[3] Haskell A. (2019). Natural history of avascular necrosis in the talus: When to operate. Foot Ankle Clin..

[4] Gross C.E., Haughom B., Chahal J., Holmes G.B. (2014). Treatments for avascular necrosis of the talus: A systematic review. Foot Ankle Spec..

[5] McGahan P.J., Pinney S.J. (2010). Current concept review: Osteochondral lesions of the talus. Foot Ankle Int..

[6] Bruns J., Habermann C., Werner M. (2021). Osteochondral lesions of the talus: A review on talus osteochondral injuries, including osteochondritis dissecans. Cartilage.

[7] Zhang H., Fletcher A.N., Scott D.J., Nunley J. (2022). Avascular osteonecrosis of the talus: Current treatment strategies. Foot Ankle Int..

[8] Hassenpflug J., Ulrich H.W., Liebs T., Lankes J.M., Terheyden H., Kreusch T., Drescher W. (2007). Vascularized iliac crest bone graft for talar defects: Case reports. Foot Ankle Int..

[9] Haddad S.L. (1998). Revision arthrodesis of the ankle and hindfoot. Foot Ankle Clin..

[10] Cifaldi A., Thompson M., Abicht B. (2022). Tibiotalocalcaneal arthrodesis with structural allograft for management of large osseous defects of the hindfoot and ankle: A systematic review and meta-analysis. J. Foot Ankle Surg..

[11] Dhillon M.S., Rana B., Panda I., Patel S., Kumar P. (2018). Management options in avascular necrosis of talus. Indian J. Orthop..

[12] DeFontes K.W., Vaughn J., Smith J., Bluman E.M. (2018). Tibiotalocalcaneal arthrodesis with bulk talar allograft for treatment of talar osteonecrosis. Foot Ankle Int..

[13] Ajis A., Tan K.J., Myerson M.S. (2013). Ankle arthrodesis vs. TTC arthrodesis: Patient outcomes, satisfaction, and return to activity. Foot Ankle Int..

[14] Franceschi F., Franceschetti E., Torre G., Papalia R., Samuelsson K., Karlsson J., Denaro V. (2016). Tibiotalocalcaneal arthrodesis using an intramedullary nail: A systematic review. Knee Surg. Sports Traumatol. Arthrosc..

[15] Shah K.S., Younger A.S. (2011). Primary tibiotalocalcaneal arthrodesis. Foot Ankle Clin..

[16] Biz C., Hoxhaj B., Aldegheri R., Iacobellis C. (2016). Minimally invasive surgery for tibiotalocalcaneal arthrodesis using a retrograde intramedullary nail: Preliminary results of an innovative modified technique. J. Foot Ankle Surg..

[17] Gross C., Erickson B.J., Adams S.B., Parekh S.G. (2015). Ankle arthrodesis after failed total ankle replacement: A systematic review of the literature. Foot Ankle Spec..

[18] Budnar V.M., Hepple S., Harries W.G., Livingstone J.A., Winson I. (2010). Tibiotalocalcaneal arthrodesis with a curved, interlocking, intramedullary nail. Foot Ankle Int..

[19] Deleu P.A., Devos Bevernage B., Maldague P., Gombault V., Leemrijse T. (2014). Arthrodesis after failed total ankle replacement. Foot Ankle Int..

[20] Escudero M.I., Poggio D., Alvarez F., Barahona M., Vivar D., Fernandez A. (2019). Tibiotalocalcaneal arthrodesis with distal tibial allograft for massive bone deficits in the ankle. Foot Ankle Surg..

[21] Jeng C.L., Campbell J.T., Tang E.Y., Cerrato R.A., Myerson M.S. (2013). Tibiotalocalcaneal arthrodesis with bulk femoral head allograft for salvage of large defects in the ankle. Foot Ankle Int..

[22] Sherman A.E., Mehta M.P., Nayak R., Mutawakkil M.Y., Ko J.H., Patel M.S., Kadakia A.R. (2022). Biologic augmentation of tibiotalocalcaneal arthrodesis with allogeneic bone block is associated with high rates of fusion. Foot Ankle Int..

[23] Rogero R., Tsai J., Fuchs D., Shakked R., Raikin S.M. (2020). Midterm results of radiographic and functional outcomes after tibiotalocalcaneal arthrodesis with bulk femoral head allograft. Foot Ankle Spec..

[24] Rogero R., Tsai J., Shakked R., Raikin S. (2018). Mid-term results of radiographic and functional outcomes after tibiotalocalcaneal arthrodesis with bulk femoral head allograft. Foot Ankle Orthop..

[25] Brachet A., Bełżek A., Furtak D., Geworgjan Z., Tulej D., Kulczycka K., Karpiński R., Maciejewski M., Baj J. (2023). Application of 3D printing in bone grafts. Cells.

[26] Kowalski C., Stauch C., Callahan R., Saloky K., Walley K., Aynardi M., Juliano P. (2020). Prognostic risk factors for complications associated with tibiotalocalcaneal arthrodesis with a nail. Foot Ankle Surg..

[27] Love B., Alexander B., Ray J., Halstrom J., Barranco H., Solar S., Singh M., Shah A. (2020). Outcomes of tibiocalcaneal arthrodesis in high-risk patients: An institutional cohort of 18 patients. Indian J. Orthop..

[28] Lee M., Choi W.J., Han S.H., Jang J., Lee J.W. (2018). Uncontrolled diabetes as a potential risk factor in tibiotalocalcaneal fusion using a retrograde intramedullary nail. Foot Ankle Surg..

[29] Pitts C., Alexander B., Washington J., Barranco H., Patel R., McGwin G., Shah A.B. (2020). Factors affecting the outcomes of tibiotalocalcaneal fusion. Bone Joint J..

[30] Wukich D.K. (2015). Diabetes and its negative impact on outcomes in orthopaedic surgery. World J. Orthop..

[31] Jiao H., Xiao E., Graves D.T. (2015). Diabetes and its effect on bone and fracture healing. Curr. Osteoporos. Rep..

[32] Benedick A., Audet M.A., Vallier H.A. (2020). The effect of obesity on post-operative complications and functional outcomes after surgical treatment of torsional ankle fracture: A matched cohort study. Injury.

[33] O’Connor K.M., Johnson J.E., McCormick J.J., Klein S.E. (2016). Clinical and operative factors related to successful revision arthrodesis in the foot and ankle. Foot Ankle Int..

[34] Perlman M.H., Thordarson D.B. (1999). Ankle fusion in a high risk population: An assessment of nonunion risk factors. Foot Ankle Int..

[35] Ahmad J., Pour A.E., Raikin S.M. (2007). The modified use of a proximal humeral locking plate for tibiotalocalcaneal arthrodesis. Foot Ankle Int..

